# Global Prevalence of Dry Eye Disease in Medical Students: A Systematic Review and Meta-Analysis

**DOI:** 10.3390/vision10030046

**Published:** 2026-07-23

**Authors:** Víctor Juan Vera-Ponce, Juan Carlos Bustamante-Rodríguez, Jhosmer Ballena-Caicedo, Leyner Fernández-Díaz, Namibia Jherly Cervera Vasquez, Fiorella E. Zuzunaga-Montoya

**Affiliations:** Facultad de Medicina (FAMED), Universidad Nacional Toribio Rodríguez de Mendoza de Amazonas (UNTRM), Chachapoyas 01001, Peru; 7103928122@untrm.edu.pe (J.C.B.-R.); 7330178022@untrm.edu.pe (J.B.-C.); 7750213231@untrm.edu.pe (L.F.-D.); 7679509541@untrm.edu.pe (N.J.C.V.); fiorellazuzunaga@untrm.edu.pe (F.E.Z.-M.)

**Keywords:** dry eye syndromes, students, medical, prevalence, systematic review, meta-analysis

## Abstract

Dry Eye Disease (DED) is a multifactorial condition increasingly affecting young populations, including medical students, due to intensive screen use, prolonged near work, and demanding academic environments. This study aimed to estimate the prevalence of DED-related outcomes among medical students and to examine how estimates vary according to diagnostic methods, methodological characteristics, and publication period. A systematic review with meta-analysis was conducted. PubMed, Scopus, Web of Science (including SciELO), and EMBASE were searched from January 2000 to March 2026. A random-effects model was applied, and subgroup, sensitivity, and exploratory meta-regression analyses were performed. Twenty-seven studies were included, comprising 12,501 participants. The pooled prevalence of DED-related outcomes was 47.4% (95% CI: 38.4–56.4%); however, between-study heterogeneity was extreme (I^2^ = 99%). Therefore, this estimate should be interpreted as an approximate summary of a highly heterogeneous evidence base rather than as a definitive global epidemiological value. Prevalence estimates varied according to diagnostic approach, particularly between self-report, symptom questionnaires, and clinical tests. Exploratory meta-regression suggested a positive association with publication year, but these analyses did not meaningfully resolve heterogeneity and should not be interpreted as causal evidence of a temporal increase. Standardized diagnostic approaches combining symptoms and ocular surface signs are needed to improve comparability across studies and inform preventive visual-health strategies in medical education.

## 1. Introduction

Dry Eye Disease (DED) is a multifactorial condition of the ocular surface characterized by loss of tear film homeostasis and ocular discomfort symptoms [1]. Epidemiological estimates vary widely because studies do not always measure the same construct. Symptom-based instruments primarily capture dry eye symptoms or symptomatic DED; sign-based tests may detect ocular surface abnormalities without necessarily confirming patient-perceived disease, and combined frameworks such as TFOS DEWS II emphasize the integration of symptoms with objective ocular surface signs [2]. Therefore, the frequently cited adult prevalence range of approximately 5% to 50% should be interpreted in light of diagnostic definition, population structure, and measurement strategy rather than as a single homogeneous epidemiological estimate [2,3].

This distinction is particularly relevant for prevalence reviews. Self-report, OSDI, DEQS, McMonnies questionnaire, Schirmer test, and other clinical approaches are not interchangeable: OSDI and DEQS mainly quantify symptom burden or quality-of-life impact, McMonnies incorporates risk-factor weighting, and Schirmer primarily assesses tear production [4,5,6,7]. Consequently, pooled estimates combining these approaches should be interpreted as DED-related outcomes unless studies apply combined symptom-and-sign definitions consistent with contemporary diagnostic recommendations.

Dry eye symptoms and DED-related outcomes have been increasingly reported among young populations, particularly university students, in association with environmental and behavioral factors [8,9]. Medical students deserve special attention because they are exposed to prolonged study periods, extensive screen and reading time, and hospital or classroom environments with air conditioning and artificial lighting that may affect the ocular surface [10,11].

Although dry eye symptoms have been increasingly reported in university populations, evidence focused specifically on medical students remains fragmented. Existing studies differ substantially in diagnostic definitions, sampling strategies, and geographic contexts, which limits direct comparability and complicates interpretation of the true burden in this group. A synthesis focused on medical students is therefore warranted, not only to estimate prevalence but also to examine how methodological decisions shape reported estimates. Therefore, this systematic review and meta-analysis aimed to estimate the prevalence of DED-related outcomes among medical students worldwide and to examine how prevalence estimates vary according to diagnostic definition, sampling approach, geographic context, and publication period.

## 2. Materials and Methods

### 2.1. Study Design

An SR with meta-analysis was conducted. For its elaboration, the PRISMA (Preferred Reporting Items for Systematic Reviews and Meta-Analyses) guidelines [12] were followed, applying relevant adaptations for prevalence studies to ensure standardized and transparent search, selection, and synthesis of evidence [13,14] (Supplementary Material S1).

The review was not registered in PROSPERO or another public registry, and no public protocol was prepared before the review began. To reduce the risk of selective analytical decisions despite the absence of preregistration, eligibility criteria, extraction variables, subgroup definitions, and statistical procedures were defined before quantitative synthesis and are reported in the manuscript and Supplementary Materials.

### 2.2. Search Strategy

A systematic search was performed in the main bibliographic databases, including MEDLINE/PubMed, Scopus, Web of Science (which incorporates the SciELO catalog), and EMBASE. The recommendations informed the selection of these databases, based on the Cochrane guidelines regarding international coverage and the relevance of these sources for clinical and epidemiological studies [15]. For the same reason, it was decided not to conduct an independent search in local databases, such as SciELO, since the Web of Science index ensured that literature from Latin America was equally covered.

To identify studies, MeSH terms and keywords related to “Dry Eye” and “Medical Students” were combined using Boolean operators (AND, OR) and delimiters according to the specific syntax of each search engine. The search period spanned from January 2000 to March 2026 to include the most recent articles and capture potential temporal patterns. The complete search strategy was documented in detail in Supplementary Material S2. Google Scholar, gray literature repositories, and manual citation tracking were not searched. The search strategy prioritized reproducible bibliographic databases with broad biomedical coverage; however, the absence of these complementary retrieval methods may have reduced the identification of unpublished studies or difficult-to-index reports.

### 2.3. Selection Criteria

Observational studies, predominantly cross-sectional, were included, although cohort designs were also considered when they provided prevalence data aligned with the study objective. Populations could be recruited through probabilistic or non-probabilistic sampling. Eligible studies defined DED-related outcomes through self-report, validated symptom questionnaires such as the Ocular Surface Disease Index (OSDI), McMonnies questionnaire, or DEQS, clinical tests such as the Schirmer test, or other recognized approaches. No language restriction was imposed, provided the full text was available and met the aforementioned criteria.

Studies that did not offer prevalence results (for example, bibliographic reviews, systematic reviews, letters to the editor), those limited to describing specific clinical cases, or those involving non-representative populations (such as students from other disciplines) were excluded. Research that did not meet clear diagnostic criteria for dry eye, or whose information was incomplete or duplicated in another previously analyzed study, was also discarded.

### 2.4. Study Selection Process

After executing the search strategy in the selected databases, the results were imported into Rayyan (Rayyan Systems Inc., Cambridge, MA, USA; web version accessed March 2026). This software facilitated the identification and removal of duplicates, ensuring that each study was evaluated only once. Subsequently, five independent reviewers examined all titles and abstracts, discarding those that did not meet the inclusion criteria.

Potentially relevant studies were then read in full text by the same five reviewers, who independently determined their definitive eligibility. Discrepancies in the selection phase were resolved through discussion, and in cases where a consensus could not be reached, a six-member review committee was consulted.

### 2.5. Data Extraction

Information from each included study was systematically extracted using a previously designed template in Excel 2023. Five researchers worked independently to extract data related to authors, year of publication, country or countries of implementation, study design, collection period, sample size, sampling method, sociodemographic characteristics of the population, applied diagnostic criteria, and prevalence results with their corresponding confidence intervals.

Each researcher cross-validated the collected information to minimize errors by comparing the extracted data and resolving discrepancies by consensus. In cases where consensus could not be reached among the five reviewers, a sixth independent reviewer was consulted to make the final decision. When inconsistencies arose with the information reported in the original article, primary sources were reviewed, or corresponding clarification was sought to ensure the fidelity of the data incorporated into the analysis.

Missing or unclear data were handled by reviewing the full text and source reports. No statistical imputation was performed; studies with unavailable numerators, denominators, or prevalence estimates were excluded from the corresponding synthesis or subgroup analysis.

### 2.6. Risk of Bias Assessment

The methodological quality and risk of bias of the included studies were evaluated using the Munn et al. tool [13], specifically designed for prevalence studies. This selection was justified by its adequacy in estimating the robustness of sampling methodologies, the definition of the condition of interest, and clarity in the presentation of results, among other aspects. Each of the domains proposed by the tool was classified according to established criteria, assigning a global level of risk of bias (low, medium, or high).

Five reviewers independently carried out the assessment, assigning scores based on the quality of sample selection, diagnostic method, and completeness of the reported information. Discrepancies were discussed among reviewers to reach a consensus. When consensus could not be achieved, a sixth independent reviewer was consulted to make the final decision. The sum of the scores and the final categorization of each study were incorporated into the synthesis of results, allowing the assessment of the possible influence of risk of bias on the global findings of the meta-analysis.

Overall risk-of-bias categories were interpreted alongside domain-level concerns, particularly sampling representativeness and outcome ascertainment. Thus, a low overall category according to the scoring system was not considered equivalent to the absence of selection bias, measurement heterogeneity, or limited external validity.

### 2.7. Statistical Analysis

Two investigators independently performed all statistical analyses using R statistical software (version 4.4.1) with the meta (version 7.0-0), metafor (version 4.6-0), ggplot2 (version 3.5.1), ggrepel (version 0.9.5), and scales (version 1.3.0) packages. A random-effects model was used to estimate prevalence, using the Freeman–Tukey double arcsine transformation to stabilize variance in proportional analyses. For the estimation of the heterogeneity parameter (τ^2^), the DerSimonian–Laird method was applied. These procedures yielded a point value for pooled prevalence, along with 95% confidence intervals and heterogeneity statistics (I^2^ and Cochran’s Q test). Results were compared between both analysts to ensure consistency, particularly regarding heterogeneity assessments, and any discrepancies were resolved by consensus.

Subgroup analyses were conducted to evaluate the impact of various factors on prevalence. Specifically, studies were grouped according to country or geographic region, sampling method (probabilistic vs. non-probabilistic), diagnostic criteria (self-report, OSDI, Schirmer test, McMonnies questionnaire, or DEQS-Th), sex, and publication period. These sub-analyses allowed the identification of possible variations in prevalence associated with methodological or contextual characteristics.

To explore small-study and reporting-bias patterns, a funnel plot was generated to evaluate the symmetry of prevalence estimates against their standard errors. Egger’s test was also applied to assess funnel plot asymmetry. Because funnel plot asymmetry in prevalence meta-analyses with extreme heterogeneity is not specific for publication bias, results were interpreted cautiously. Sensitivity analyses were carried out by excluding the study with the largest sample size and by excluding studies with the lowest and highest prevalence estimates to examine the robustness of the pooled estimate.

Finally, random-effects meta-regression models were performed to evaluate the effect of potential moderating variables, including publication year, sample size, diagnostic method, sampling design, geographic region, and the percentage of women in the sample. Meta-regression analyses were considered exploratory because the number of included studies was limited relative to the number of potential moderators, and publication year was assessed using the year reported in the final reference list.

## 3. Results

### 3.1. Article Selection Flow

**Figure 1:**
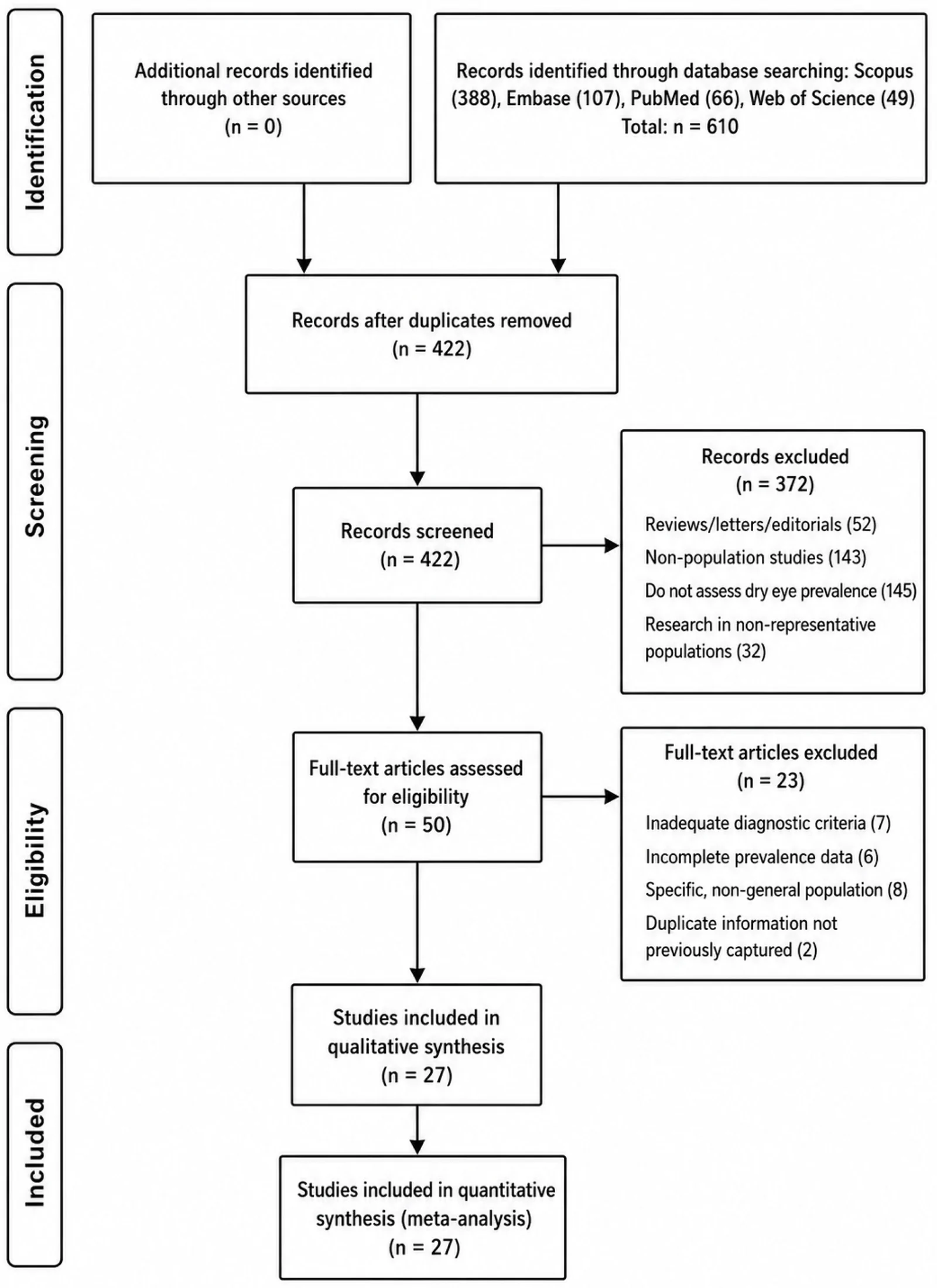
Flowchart of Study Selection.

Searches in the databases above identified 610 references (Figure 1). After removing duplicates, 422 records remained for the selection phase, of which 372 were excluded because they did not meet the eligibility criteria (including review articles, non-representative populations, and studies that did not evaluate prevalence). Of the 50 full texts evaluated, 23 were excluded: seven because of inadequate diagnostic criteria, six because of incomplete prevalence data, eight because they described specific, non-general populations, and two because they contained duplicate information not previously captured. Finally, 27 studies met all requirements and were included in the qualitative synthesis and meta-analysis [16,17,18,19,20,21,22,23,24,25,26,27,28,29,30,31,32,33,34,35,36,37,38,39,40,41,42].

### 3.2. General Characteristics of Included Studies

The 27 selected studies were published between 2019 and 2025 (Supplementary Material S3). Specifically, two studies (7.4%) corresponded to 2019, three (11.1%) to 2020, two (7.4%) to 2021, six (22.2%) to 2022, seven (25.9%) to 2023, six (22.2%) to 2024, and one (3.7%) to 2025. The highest concentration of publications was observed in 2023, followed by 2022 and 2024. Regarding geographical distribution, the studies came from 13 countries, with India standing out with seven studies [16,19,21,27,29,33,34], Nepal (3) [23,26,38], Jordan (3) [25,36,41], Saudi Arabia (3) [18,37,42], and Thailand (2) [28,40]. Research from Pakistan [17], Romania [39], Serbia [24], Sudan [31], and Iraq [20] was also included, as well as a Latin American multinational study [30] and another conducted independently in Peru [22]. Although the language of publication was not specified in most cases, it was observed that the majority were available in English.

Regarding sampling, 25 studies (92.6%) employed non-probabilistic methods (convenience or volunteer) [16,17,18,19,20,21,22,23,24,25,26,27,28,29,30,32,33,34,35,36,37,38,39,40,41], while two studies (7.4%) applied probabilistic sampling [31,42]. Sample sizes varied between 110 (the smallest) and 2429 participants (the largest), with a median of 274 (range 110–2429) and an approximate average of 463 participants. Most studies used questionnaires to collect data for sociodemographic characteristics and the evaluation of dry eye symptoms.

The included populations primarily comprised medical students aged, in most cases, between 18 and 26 years, although some studies reached ranges up to 30 or even 38 years. Regarding sex distribution, the percentage of women ranged approximately between 36.9% and 81.4%, with no studies reported as exclusively directed at one sex. Some works specified the inclusion of students from different courses (from first year to internship), and, in several cases, participants with systemic ocular diseases, recent surgery history, or use of medications with potential influence on the ocular surface were excluded.

Regarding risk of bias, the greatest domain-level concerns were related to non-probabilistic sampling and heterogeneous outcome ascertainment. One study (Aljammaz, 2023) [42] reached 9 points by meeting both probabilistic sampling and validated-method criteria; 16 studies obtained 8 points, and the remaining 10 obtained 7 points. According to the predefined scoring approach, all studies reached the low overall risk-of-bias category; however, this global classification should be interpreted cautiously because non-probability sampling and heterogeneous outcome ascertainment were frequent domain-level concerns.

The funnel plot (Freeman–Tukey arcsine transformation) showed wide dispersion and asymmetry. However, in prevalence meta-analyses with extreme between-study heterogeneity, funnel plot asymmetry should not be interpreted as direct evidence of publication bias. It may also reflect true differences in prevalence, diagnostic heterogeneity, sampling variability, or instability of variance estimates. Therefore, the observed asymmetry was interpreted as evidence of small-study and methodological effects rather than as a specific indicator of missing unpublished studies (Supplementary Material S4).

### 3.3. Meta-Analysis of DED-Related Outcomes

**Figure 2:**
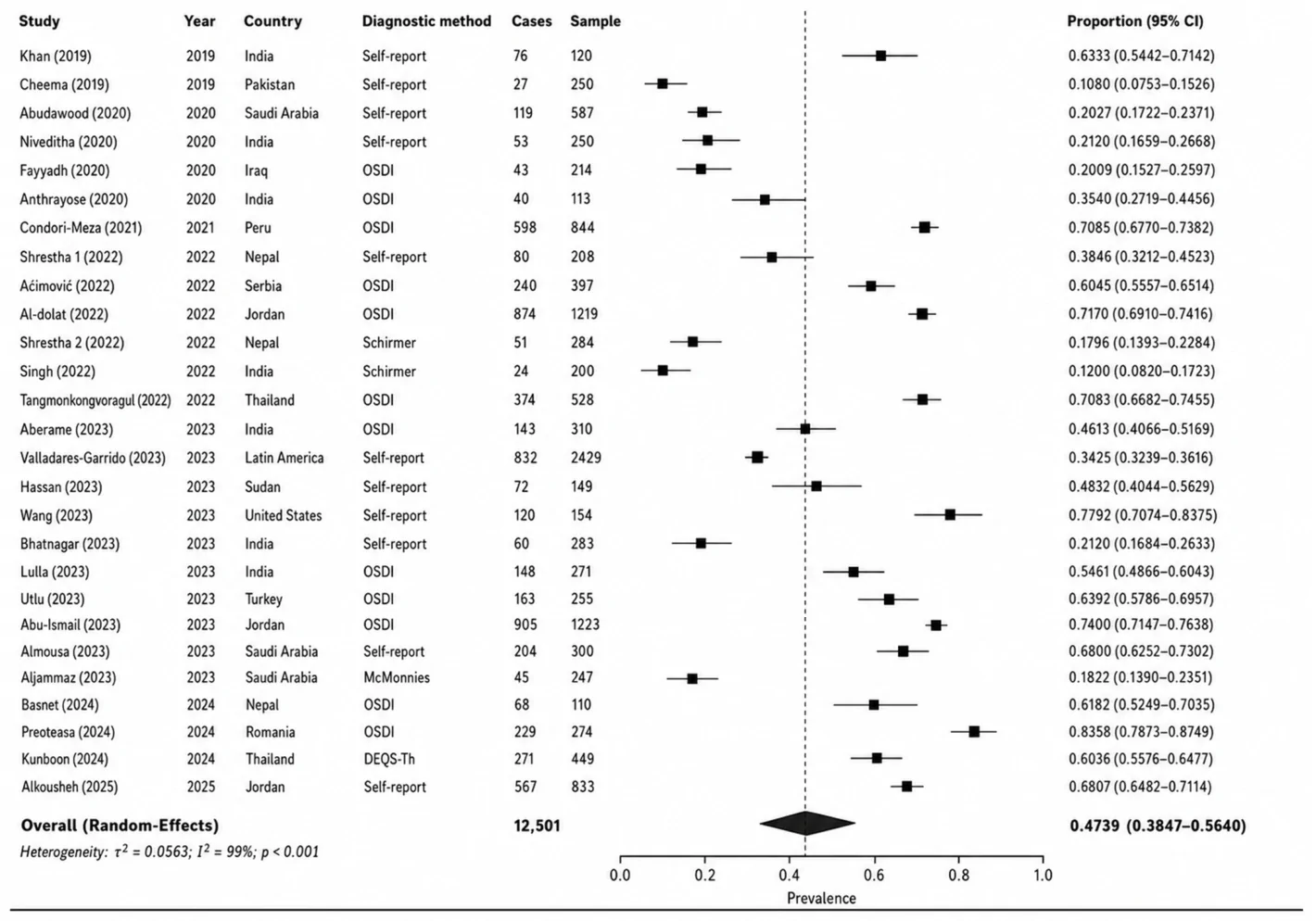
Meta-analysis of DED-related outcomes in medical students. Labels correspond to included studies [16,17,18,19,20,21,22,23,24,25,26,27,28,29,30,31,32,33,34,35,36,37,38,39,40,41,42]; squares, lines, diamond, and dashed line denote study estimates, 95% CIs, pooled estimate, and pooled prevalence.

Twenty-seven studies were included in the meta-analysis, with a cumulative total of 12,501 participants (Figure 2). Using a random-effects model, the pooled prevalence of DED-related outcomes among medical students was 47.4% (95% CI: 38.4–56.4%). Extreme heterogeneity was observed among studies (I^2^ = 99%, *p* < 0.0001), with individual proportions ranging from 10.8% (95% CI: 7.5–15.3%) to 83.6% (95% CI: 78.7–87.9%). Therefore, the pooled estimate should be interpreted as an approximate summary of heterogeneous DED-related outcomes rather than as a definitive global epidemiological value. The two lowest prevalences corresponded to Cheema (2019) [17], with 10.8% in Pakistan, and Singh (2022) [27], with 12.0% in India. At the opposite extreme, the highest proportions were observed in Preoteasa (2024) [39], with 83.6% in Romania, and Wang (2023) [32], with 77.9% in the United States.

### 3.4. Subgroup and Sensitivity Analyses of DED-Related Outcomes

Table 1 summarizes subgroup analyses according to sampling design, diagnostic criteria, sex, and country. Studies using non-probabilistic sampling (*n* = 25; 12,105 participants) showed a pooled prevalence of 48.62% (95% CI: 39.38–57.91%; I^2^ = 99.0%), whereas probabilistic studies (*n* = 2; 396 participants) showed a pooled prevalence of 32.32% (95% CI: 7.91–63.71%; I^2^ = 97.5%).

Prevalence estimates varied substantially by diagnostic approach. Self-report studies (*n* = 11; 5563 participants) showed a pooled prevalence of 42.00% (95% CI: 29.34–55.23%; I^2^ = 98.8%), while OSDI-based studies (*n* = 12; 5758 participants) showed a higher pooled prevalence of 59.99% (95% CI: 51.69–68.02%; I^2^ = 97.4%). Studies using Schirmer test alone (*n* = 2; 484 participants) showed a lower pooled prevalence of 15.17% (95% CI: 9.86–21.38%; I^2^ = 68.7%). Single-study estimates were 18.22% (95% CI: 13.90–23.51%) for the McMonnies questionnaire and 60.36% (95% CI: 55.76–64.77%) for DEQS-Th.

Sex-stratified data were available for 12 studies. The pooled prevalence was 45.97% among women (95% CI: 31.05–61.27%; I^2^ = 98.9%) and 39.85% among men (95% CI: 28.53–51.73%; I^2^ = 97.1%). Although point estimates were higher among women, between-study heterogeneity remained extreme and analyses were based on aggregate study-level data; therefore, sex-specific estimates should be interpreted cautiously.

Country-level estimates should be interpreted cautiously because several countries were represented by a single study. Among countries with at least two studies, pooled prevalence estimates were 35.21% for India, 34.35% for Saudi Arabia, 38.42% for Nepal, 71.36% for Jordan, and 65.69% for Thailand, with substantial residual heterogeneity in most subgroups. Apparent geographic differences may therefore reflect diagnostic instruments, recruitment procedures, sampling frames, or study period rather than true national differences in DED prevalence. Leave-one-out and extreme-study sensitivity analyses showed that the pooled estimate was not driven by a single study. Excluding the largest study yielded a pooled prevalence of 47.92% (95% CI: 38.58–57.32%), whereas excluding the lowest and highest prevalence studies yielded estimates of 49.02% and 45.89%, respectively. However, heterogeneity remained extreme in all analyses (I^2^ ≈ 99%), indicating that sensitivity analyses did not resolve the underlying clinical and methodological variability.

### 3.5. Exploratory Meta-Regression of DED-Related Outcomes

**Figure 3:**
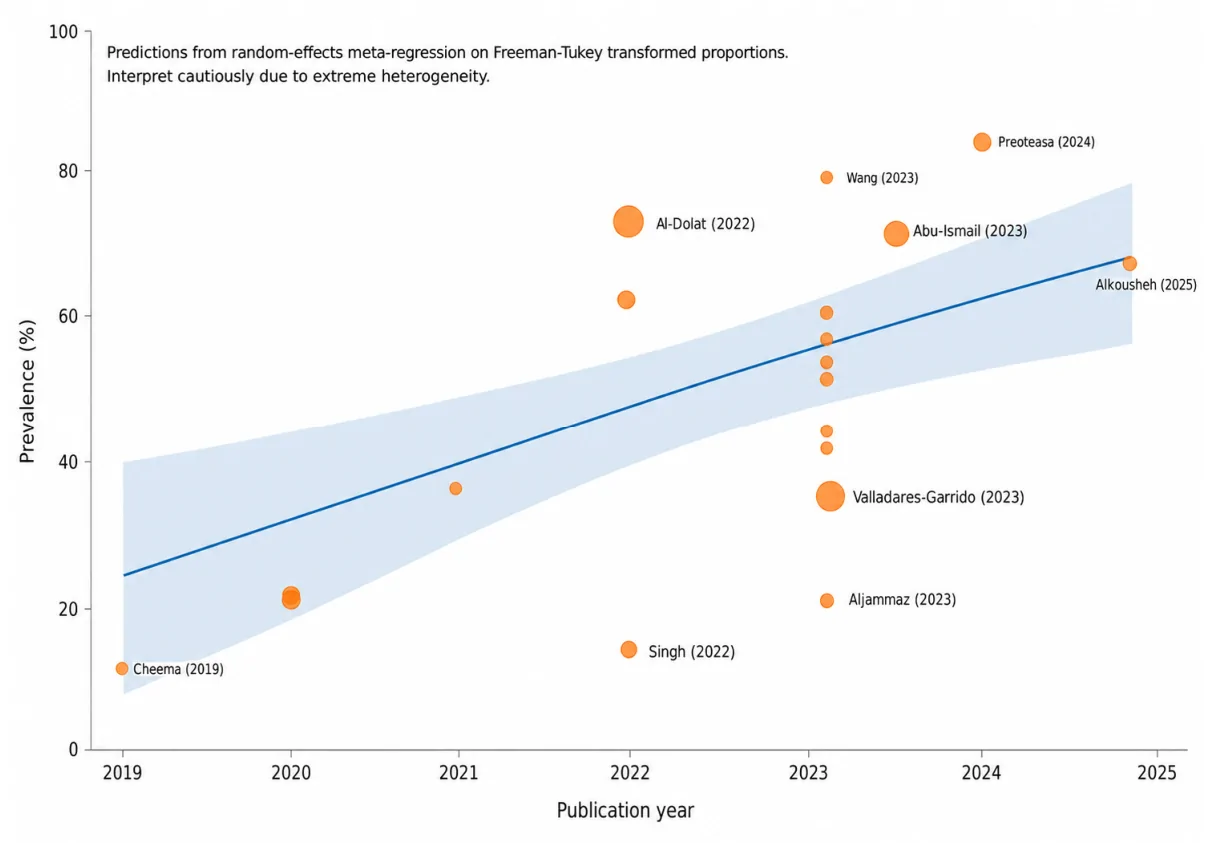
Exploratory meta-regression of DED-related outcomes according to publication year. Labels correspond to included studies [16,17,18,19,20,21,22,23,24,25,26,27,28,29,30,31,32,33,34,35,36,37,38,39,40,41,42]; the blue line shows the back-transformed random-effects meta-regression prediction and the shaded area shows the 95% CI. Interpret cautiously due to extreme residual heterogeneity.

Regarding the exploratory meta-regression analysis (Figure 3), the variables assessed as possible predictors were publication year, sample size, diagnostic method, sampling design, geographic region, and the percentage of women in the sample. Publication year was based on the final reference list and centered before analysis. Random-effects meta-regression models used the same transformed effect measure and variance structure as the main meta-analysis.

Exploratory random-effects meta-regression showed a positive association between publication year and DED-related prevalence estimates. However, residual heterogeneity remained high, and the number of included studies was limited relative to the number of potential moderators. Diagnostic method also contributed to between-study variability, with OSDI-based studies tending to report higher prevalence than self-report studies, whereas sampling design and sample size did not meaningfully explain heterogeneity. These findings should therefore be interpreted as hypothesis-generating rather than as evidence of a causal temporal increase.

## 4. Discussion

### 4.1. Comparison with Existing Literature

The pooled estimate suggests that DED-related outcomes are frequent among medical students, with a magnitude comparable to that reported in other university populations. For example, studies of university students have reported high frequencies of dry eye symptoms in South America [43], and virtual education during the pandemic was associated with a high burden of ocular symptoms in university cohorts [40,44]. However, these comparisons should be interpreted cautiously because symptom-based estimates, clinically confirmed DED, and DED-related screening outcomes are not directly interchangeable.

Compared with young adults in the general population, the estimates observed in medical students appear high. In working young and middle-aged adults, lower estimates have often been reported; for example, an office-worker study in China found a 40.3% prevalence of clinical dry eye [8], and a large population-based cohort in the Netherlands reported approximately 9.1% overall dry eye prevalence using standardized questionnaires [45]. Nevertheless, direct comparison remains limited by differences in diagnostic definitions, sampling frames, and exposure profiles.

Traditionally, DED has been associated with older adults, but the available evidence suggests that young medical students may also present a substantial burden of DED-related symptoms. Population studies indicate that approximately 10–20% of people over 40 years may present moderate to severe dry eye symptoms or seek treatment for this condition [46], although higher estimates have been described in specific regions and populations [43]. The present findings therefore reinforce that age is not the only determinant of DED-related symptom burden.

The meta-regression should be interpreted as exploratory. A positive association with publication year was observed, but extreme residual heterogeneity, the short publication window, and the coexistence of diagnostic, geographic, and sampling differences prevent causal or definitive temporal inference. The observed pattern is compatible with changes in digital exposure and educational environments, but it cannot establish a true secular increase in DED among medical students.

### 4.2. Explanation of the Observed Variations

The higher prevalence observed in recent studies may be partly related to intensive digital screen use, reduced blink rate, incomplete blinking, and environmental conditions that destabilize the tear film. However, because most included studies were cross-sectional, these mechanisms provide biological plausibility rather than evidence of causality [2,47,48,49,50,51,52].

The COVID-19 pandemic and the expansion of online education may have intensified exposure to digital devices and indoor environments. Nevertheless, our meta-regression did not establish that pandemic-related exposure caused the observed pattern. The post-2019 distribution should therefore be interpreted as a descriptive trend compatible with pandemic-related behavioral changes, not as causal evidence [9,44]. Poor sleep quality, academic stress, reduced outdoor activities, enclosed environments with air conditioning, and mask use may also contribute to ocular surface symptoms, but these associations require longitudinal and standardized assessment before causal conclusions can be drawn [36,40].

Geographical variability may explain part of the differences observed between studies, but country-level comparisons should not be overinterpreted. Several countries were represented by only one study, and apparent differences may reflect diagnostic tools, sampling procedures, environmental exposures, or study period rather than true national prevalence. This limitation is particularly relevant when comparing estimates across Asia, Latin America, Europe, and the Middle East, where population structure and measurement strategies differ substantially [53,54,55].

Differences in the diagnostic method employed constitute one of the strongest explanations for the variability between studies. Symptom-based instruments such as OSDI, DEQS, McMonnies, and simple self-report tend to yield different estimates than clinical tests such as Schirmer. OSDI focuses on symptom frequency and functional impact [4], McMonnies incorporates risk-factor weighting [5], DEQS includes quality-of-life impact [6], and self-report often lacks validation. Therefore, these instruments should not be treated as interchangeable measurements of clinically confirmed DED.

The reliance on objective tests such as the Schirmer test as standalone diagnostic criteria also presents methodological concerns. In our subgroup analysis, studies using Schirmer test alone reported a lower pooled prevalence of 15.17% (95% CI: 9.86–21.38%), which may reflect under-detection of symptomatic cases with normal tear production or a narrow sign-based definition. Conversely, isolated signs may identify ocular surface abnormalities without necessarily confirming patient-perceived disease. Recent guidelines, such as TFOS DEWS II [2], recommend combining symptoms with objective signs to improve diagnostic accuracy. This methodological heterogeneity makes direct comparability difficult and underscores the need for standardized diagnostic criteria in prevalence studies.

Sex-stratified analyses suggested higher point estimates among women than men; however, these results should not be interpreted as definitive evidence of an individual-level sex effect. The analysis was restricted to studies reporting sex-specific events and denominators, relied on aggregate data, and remained highly heterogeneous. Therefore, the findings are compatible with the broader literature identifying female sex as a risk factor for DED, but they cannot establish an independent association in medical students without individual participant data and adjustment for diagnostic method, screen exposure, contact lens use, and other confounders [46].

Overall, methodological heterogeneity remains the central interpretive constraint of this review. The aggregation of divergent definitions under a single pooled estimate provides a general reference point, but it inevitably compromises clinical interpretability. For this reason, the pooled estimate should be read together with the diagnostic subgroup analyses rather than as a standalone global prevalence. Future epidemiological studies should prioritize standardized, combined symptom-and-sign definitions to improve comparability and certainty.

### 4.3. Implications of the Findings for Education and Public Health

The findings support the inclusion of visual-health education within medical training, but the implications should be framed as preventive and educational rather than causal. Medical students could benefit from awareness of DED pathophysiology, risk factors, ergonomic practices, and early symptom recognition, both for their own ocular health and for future clinical practice.

Medical schools and universities could consider pragmatic prevention strategies, including visual ergonomics education, regular screen breaks, optimization of lighting and humidity, and early referral pathways for students with persistent symptoms. These interventions are biologically plausible and low risk, although their effectiveness in this population should be tested in prospective studies.

DED-related symptoms may affect quality of life, concentration, and academic functioning. However, the magnitude of these effects in medical students requires standardized outcome measurement. Future research should therefore evaluate both ocular outcomes and educational or quality-of-life consequences using longitudinal designs.

### 4.4. Strengths and Limitations

This systematic review has several strengths, including a broad search across major biomedical databases, clearly defined eligibility criteria focused on medical students, duplicate study selection and extraction, standardized risk-of-bias assessment, and quantitative synthesis with subgroup, sensitivity, and exploratory meta-regression analyses. The reanalysis also corrected diagnostic subgroup coding and sex-stratified estimates, improving internal consistency of the results.

The main limitation of this review is not merely statistical heterogeneity, but clinical and methodological non-equivalence across included studies. Prevalence estimates were derived from self-report, symptom questionnaires, other screening tools, and clinical tests, which do not capture identical constructs; consequently, the pooled estimate should be interpreted as an approximate summary of DED-related outcomes rather than a definitive estimate of clinically confirmed DED. In addition, most studies used non-probability sampling, limiting external validity and increasing susceptibility to selection bias. The review was not preregistered, did not include Google Scholar, gray literature, or citation tracking, and did not apply a formal certainty-of-evidence framework; these factors reduce confidence in the evidence base. Finally, sex analyses relied on aggregate data and remained highly heterogeneous, preventing individual-level inference.

## 5. Conclusions

DED-related symptoms and diagnoses appear frequent among medical students, but the pooled estimate of 47.4% should be interpreted cautiously because of extreme heterogeneity, non-probability sampling, and non-equivalent diagnostic definitions. The evidence supports the need for standardized epidemiological definitions combining symptom assessment with ocular surface signs, as well as preventive visual-health strategies in medical schools. However, temporal patterns and associations with digital exposure should be considered hypothesis-generating until confirmed by standardized longitudinal studies.

In practical terms, medical schools may consider integrating visual ergonomics, regular visual rest, periodic ophthalmological assessment for persistent symptoms, and education on ocular surface health into student wellness programs. Future research should use harmonized TFOS DEWS II-compatible definitions, probabilistic sampling when feasible, and longitudinal designs capable of separating diagnostic-method effects from true epidemiological variation.

## Figures and Tables

**Table 1:** Subgroup and sensitivity analysis of the prevalence of DED-related outcomes in medical students according to sampling design, diagnostic criteria, sex, and country.

	Number of Studies	Number of Participants	Prevalence % (95% CI)	I^2^
**Sampling design**				
Non-probabilistic	25	12,105	48.62 (39.38–57.91)	99.0%
Probabilistic	2	396	32.32 (7.91–63.71)	97.5%
**Diagnostic criteria**				
Self-report	11	5563	42.00 (29.34–55.23)	98.8%
OSDI	12	5758	59.99 (51.69–68.02)	97.4%
Schirmer test	2	484	15.17 (9.86–21.38)	68.7%
McMonnies questionnaire	1	247	18.22 (13.90–23.51)	NA
DEQS-Th	1	449	60.36 (55.76–64.77)	NA
**Sex**				
Women	12	4131	45.97 (31.05–61.27)	98.9%
Men	12	2851	39.85 (28.53–51.73)	97.1%
**Country**				
India	7	1547	35.21 (21.87–49.85)	97.1%
Pakistan	1	250	10.80 (7.53–15.26)	NA
Saudi Arabia	3	1134	34.35 (8.85–66.21)	99.1%
Iraq	1	214	20.09 (15.27–25.97)	NA
Peru	1	844	70.85 (67.70–73.82)	NA
Nepal	3	602	38.42 (16.42–63.29)	97.3%
Serbia	1	397	60.45 (55.57–65.14)	NA
Jordan	3	3275	71.36 (68.10–74.52)	76.5%
Thailand	2	977	65.69 (55.14–75.52)	91.6%
Latin America	1	2429	34.25 (32.39–36.16)	NA
Sudan	1	149	48.32 (40.44–56.29)	NA
United States	1	154	77.92 (70.74–83.75)	NA
Turkey	1	255	63.92 (57.86–69.57)	NA
Romania	1	274	83.58 (78.73–87.49)	NA

CI = confidence interval; I^2^ = percentage of total variation due to heterogeneity; NA = not applicable for single-study subgroups.

## Data Availability

The original contributions presented in this study are included in the article/Supplementary Material. Further inquiries can be directed to the corresponding author.

## Supplementary Materials

The following supporting information can be downloaded at: https://www.mdpi.com/article/10.3390/vision10030046/s1, Supplementary Material S1: PRISMA checklist adapted for SR of prevalence; Supplementary Material S2: Search strategy; Supplementary Material S3: Main characteristics of the selected studies; Supplementary Material S4: Funnel plot of the prevalence of dry eye disease in medical students.
