# Supplementary material for: Global Prevalence of Dry Eye Disease in Medical Students: A Systematic Review and Meta-Analysis
Source: Vision (Basel). 2026 Jul 23;10(3):46. doi: 10.3390/vision10030046 (PMC13517965; doi:10.3390/vision10030046)
Supplement: Supplementary file 1 [file vision-10-00046-s001.zip › DryEye_SM4_Funnel_Plot.pdf]

Supplementary Material S4. Funnel plot of DED-related outcomes in medical students

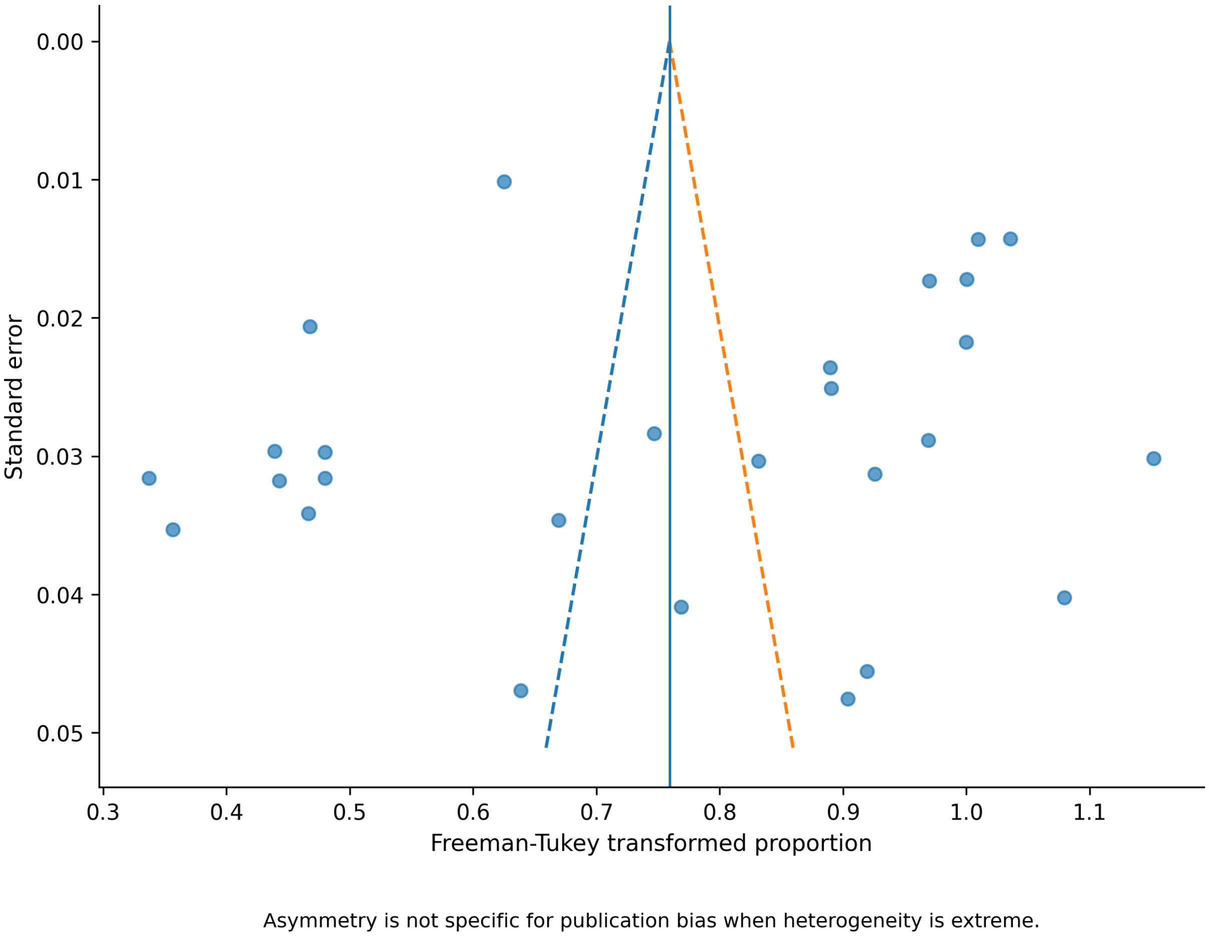

Figure S4. Funnel plot using Freeman-Tukey transformed proportions. The dashed pseudo-confidence limits are shown for visual assessment only; asymmetry is not specific for publication bias when between-study heterogeneity is extreme.
