# Supplementary material for: Global Prevalence of Dry Eye Disease in Medical Students: A Systematic Review and Meta-Analysis
Source: Vision (Basel). 2026 Jul 23;10(3):46. doi: 10.3390/vision10030046 (PMC13517965; doi:10.3390/vision10030046)
Supplement: Supplementary file 1 [file vision-10-00046-s001.zip › DryEye_SM1_PRISMA_Checklist.pdf]

Supplementary Material S1. PRISMA checklist adapted for systematic reviews of prevalence

No.	Recommendation	Location in the manuscript / response
1	Title: Identify the report as a systematic review.	Title: "Global Prevalence of Dry Eye Disease in Medical Students: A Systematic Review and Meta-Analysis."
2	Structured abstract and registration information.	Abstract: revised to report 27 studies, 12,501 participants, pooled prevalence of DED-related outcomes of 47.4% (95% CI: 38.4-56.4%), extreme heterogeneity (I2=99%), diagnostic heterogeneity, and exploratory meta-regression. Registration: the review was not registered in PROSPERO or another public registry.
3	Rationale: Describe the rationale in the context of existing knowledge.	Introduction, paragraphs 1-3: revised to contextualize adult prevalence estimates according to symptom-based definitions, sign-based definitions, combined TFOS DEWS II criteria, and the specific vulnerability of medical students.
4	Objectives: Provide an explicit statement of the objective or question.	Introduction, final paragraph: revised objective states that the review aimed to estimate DED-related outcomes among medical students and examine variation according to diagnostic definition, sampling approach, geographic context, and publication period.
5	Eligibility criteria and grouping of studies.	Methods, Selection criteria: observational studies among medical students reporting prevalence of dry eye symptoms, symptomatic DED, or clinically assessed DED were eligible. Studies were grouped by sampling design, diagnostic criterion, country, sex, and publication period when data were available.
6	Information sources and dates searched.	Methods, Search strategy: MEDLINE/PubMed, Scopus, Web of Science including the SciELO collection, and EMBASE were searched for January 2000-March 2026; the last search was performed in March 2026. Google Scholar, gray literature repositories, and manual citation tracking were not searched.
7	Full search strategies for all databases.	Methods and Supplementary Material 2: complete database-specific search strings are provided. The revised manuscript also states that the absence of Google Scholar, gray literature, and citation tracking may have reduced retrieval of unpublished or difficult-to-index reports.
8	Selection process.	Methods, Study selection process: records were imported into Rayyan; five independent reviewers screened titles/abstracts and full texts. Disagreements were solved by consensus or by a sixth reviewer.
9	Data collection process.	Methods, Data extraction: a predefined Excel 2023 template was used; five reviewers extracted data independently, cross-validated the extraction, and consulted a sixth reviewer if consensus was not reached.
10	Data items and handling of missing or unclear information.	Methods, Data extraction: extracted variables included authors, year, country, design, sampling, sample size, sex, age, diagnostic criterion, events, denominators, and prevalence estimates. No statistical imputation was performed; studies with unavailable data were excluded from the corresponding synthesis or subgroup analysis.
11	Study risk-of-bias assessment.	Methods, Risk of bias assessment: the Munn et al. tool was applied independently by five reviewers. The revised manuscript clarifies that low overall risk-of-bias scores were interpreted alongside domain-level concerns, especially non-probability sampling and heterogeneous outcome ascertainment.
12	Effect measures.	Methods, Statistical analysis: the effect measure was the prevalence/proportion, using the Freeman-Tukey double arcsine transformation for variance stabilization in proportional meta-analysis.
13	Synthesis methods, software, heterogeneity exploration, and sensitivity analyses.	Methods, Statistical analysis: random-effects models using the DerSimonian-Laird estimator for tau2 were applied. Subgroup analyses examined sampling design, diagnostic criterion, country, sex, and publication period. Meta-regression models were considered exploratory. Analyses were performed in R using the meta, metafor, ggplot2, ggrepel, and scales packages; analytic code and session information are available upon reasonable request.
14	Reporting bias assessment.	Methods, Statistical analysis and Results: funnel plot and Egger-type asymmetry assessment were used, but the revised manuscript emphasizes that asymmetry is not specific for publication bias in prevalence meta-analyses with extreme heterogeneity.
15	Certainty assessment.	Methods/Discussion: no formal GRADE or equivalent certainty-of-evidence framework was applied. The revised Discussion explicitly recognizes this as a limitation affecting confidence in the evidence base.
16	Study selection results.	Results, Article selection flow and Figure 1: 610 references identified, 422 after duplicate removal, 50 full texts assessed, and 27 studies included in the qualitative and quantitative synthesis.
17	Study characteristics.	Results and Supplementary Material 3: study years were harmonized to publication year: 2 studies in 2019, 3 in 2020, 2 in 2021, 6 in 2022, 7 in 2023, and 7 in 2024. The revised table also reports diagnostic method and event/sample data.
18	Risk of bias in studies.	Results, General characteristics and Supplementary Material 3: all studies reached low overall risk-of-bias category under the predefined score; however, the revised text highlights non-probability sampling and heterogeneous outcome ascertainment as frequent domain-level concerns.
19	Results of individual studies.	Results, Figure 2 and Supplementary Material 3: individual estimates ranged from 10.8% in Cheema (2019) to 83.6% in Preoteasa (2024), with events, denominators, and diagnostic method shown in the revised study characteristics table.

No.	Recommendation	Location in the manuscript / response
20	Results of syntheses and investigations of heterogeneity.	Results, Table 1: corrected subgroup analyses report 25 non-probabilistic and 2 probabilistic studies; 11 self-report, 12 OSDI, 2 Schirmer test, 1 McMonnies, and 1 DEQS-Th study; and corrected sex-specific estimates based on 12 studies.
21	Reporting biases.	Results and Supplementary Material 4: the funnel plot was replaced with a cleaner version and interpreted as evidence of small-study/methodological effects rather than direct proof of publication bias.
22	Certainty of evidence.	Not applicable: no formal GRADE assessment was performed. The limitation is now explicitly stated in the revised manuscript.
23	Discussion of findings, limitations, and implications.	Discussion: revised to frame the pooled prevalence as an approximate summary of heterogeneous DED-related outcomes, to moderate causal language about screen exposure and COVID-19, and to integrate limitations related to non-probability sampling, diagnostic non-equivalence, no preregistration, and no gray-literature search.
24	Registration and protocol.	Methods and PRISMA item: the review was not registered in PROSPERO or another registry, and no public protocol was prepared before the review began.
25	Support.	Funding statement: the study was funded by the Vicerectorado de Investigación of the Universidad Nacional Toribio Rodríguez de Mendoza de Amazonas, with no role in study design, data collection, analysis, interpretation, or manuscript preparation.
26	Competing interests.	Conflict of Interest statement: the authors declare no conflict of interest.
27	Availability of data, code, and other materials.	Data Availability Statement: all data generated or analyzed during this systematic review and meta-analysis are included in the article and supplementary materials; extracted data and analytic code are available upon reasonable request from the corresponding author.
