# Supplementary material for: Global Prevalence of Dry Eye Disease in Medical Students: A Systematic Review and Meta-Analysis
Source: Vision (Basel). 2026 Jul 23;10(3):46. doi: 10.3390/vision10030046 (PMC13517965; doi:10.3390/vision10030046)
Supplement: Supplementary file 1 [file vision-10-00046-s001.zip › DryEye_SM2_Search_Strategy.pdf]

Supplementary Material S2. Search strategy

Database	Set	Search string
PubMed	#1	"Dry Eye Syndromes"[Mesh] OR "dry eye syndrome"[tiab] OR "dry eye syndromes"[tiab] OR "dry eyes"[tiab] OR "ojo seco"[tiab] OR "ojos secos"[tiab] OR "ocular dryness"[tiab] OR xerophthalmia[tiab]
	#2	"Students"[Mesh] OR "Students, Medical"[Mesh] OR "medical student"[tiab] OR "medical students"[tiab] OR "college student"[tiab] OR "college students"[tiab] OR "university student"[tiab] OR "university students"[tiab] OR "undergraduate student"[tiab] OR "undergraduate students"[tiab]
	#3	#1 AND #2
Scopus	#1	TITLE-ABS-KEY ("Dry Eye Syndromes" OR "dry eye syndrome" OR "dry eye syndromes" OR "dry eyes" OR "ojo seco" OR "ojos secos" OR "ocular dryness" OR xerophthalmia)
	#2	TITLE-ABS-KEY ("medical student" OR "medical students" OR "college student" OR "college students" OR "university student" OR "university students" OR "undergraduate student" OR "undergraduate students" OR "students, medical" OR "students")
	#3	#1 AND #2
Web of Science	#1	TS=("Dry Eye Syndromes" OR "dry eye syndrome" OR "dry eye syndromes" OR "dry eyes" OR "ojo seco" OR "ojos secos" OR "ocular dryness" OR xerophthalmia)
	#2	TS=("medical student" OR "medical students" OR "college student" OR "college students" OR "university student" OR "university students" OR "undergraduate student" OR "undergraduate students" OR "students, medical" OR "students")
	#3	#1 AND #2
EMBASE	#1	'dry eye syndrome'/exp OR 'dry eye syndrome' OR 'dry eye syndromes' OR 'dysfunctional tear syndrome'
	#2	'student'/exp OR 'medical student'/exp OR 'medical student':ti,ab OR 'medical students':ti,ab OR 'college student':ti,ab OR 'college students':ti,ab OR 'university student':ti,ab OR 'university students':ti,ab OR 'undergraduate student':ti,ab OR 'undergraduate students':ti,ab
	#3	#1 AND #2
