# Supplementary material for: Global Prevalence of Dry Eye Disease in Medical Students: A Systematic Review and Meta-Analysis
Source: Vision (Basel). 2026 Jul 23;10(3):46. doi: 10.3390/vision10030046 (PMC13517965; doi:10.3390/vision10030046)
Supplement: Supplementary file 1 [file vision-10-00046-s001.zip › DryEye_SM3_Study_Characteristics.pdf]

Supplementary Material S3. Main characteristics of the selected studies

Study	Year	Country	Sampling	Diagnostic method	DED cases/sample	Prevalence	Sex (% female)	Age	Overall risk of bias	Selection criteria
Khan (2019)	2019	India	Non-probabilistic	Self-report	76/120	63.33%	59 (50.0%)	21.55	7 (Low)	Medical students.
Cheema (2019)	2019	Pakistan	Non-probabilistic	Self-report	27/250	10.80%	148 (59.2%)	23	7 (Low)	Medical students aged 21-25 years using digital devices; exclusions included ocular/systemic conditions, medication affecting vision, and no consent.
Abudawood (2020)	2020	Saudi Arabia	Non-probabilistic	Self-report	119/587	20.27%	319 (54.3%)	21.65	7 (Low)	Medical students using computers/tablets for at least one month.
Niveditha (2020)	2020	India	Non-probabilistic	Self-report	53/250	21.20%	128 (51.2%)	Not reported	7 (Low)	Medical students; non-medical students excluded.
Fayyadh (2020)	2020	Iraq	Non-probabilistic	OSDI	43/214	20.09%	117 (54.7%)	20.95	8 (Low)	Medical students.
Anthrayose (2021)	2021	India	Non-probabilistic	OSDI	40/113	35.40%	84 (74.3%)	22.27	8 (Low)	Medical students with consent and complete questionnaire.
Condori-Meza (2022)	2022	Peru	Non-probabilistic	OSDI	598/844	70.85%	543 (64.3%)	21.8	8 (Low)	Medical students older than 18 years; exclusions included Sjogren syndrome, refractive surgery, glaucoma, uncorrected visual problems, and psychiatric disease.
Shrestha 1 (2023)	2023	Nepal	Non-probabilistic	Self-report	80/208	38.46%	80 (38.5%)	22.7	7 (Low)	First- to fourth-year Medicine and Surgery students.
Aćimović (2022)	2022	Serbia	Non-probabilistic	OSDI	240/397	60.45%	301 (75.8%)	22.6	8 (Low)	Medical students.
Al-Dolat (2022)	2022	Jordan	Non-probabilistic	OSDI	874/1219	71.70%	711 (58.3%)	Not reported	8 (Low)	First- to sixth-year medical students.
Shrestha 2 (2022)	2022	Nepal	Non-probabilistic	Schirmer test	51/284	17.96%	105 (36.97%)	22.8	8 (Low)	Medical students with consent and complete questionnaire.
Singh (2022)	2022	India	Non-probabilistic	Schirmer test	24/200	12.00%	109 (54.5%)	21	8 (Low)	First- to fifth-year medical students aged 18-25 years; history of eye disease excluded.
Tangmonkongvoragul (2022)	2022	Thailand	Non-probabilistic	OSDI	374/528	70.83%	276 (52.3%)	20.48	8 (Low)	Medical students with completed questionnaire and examination; ocular trauma/surgery, ocular disease, or relevant medication use excluded.
Aberame (2023)	2023	India	Non-probabilistic	OSDI	143/310	46.13%	181 (58.4%)	Not reported	8 (Low)	Medical students aged 18-26 years; non-consent, ocular/systemic disease, and medication use excluded.
Valladares-Garrido (2023)	2023	Latin America	Non-probabilistic	Self-report	832/2429	34.25%	1259 (51.8%)	23	7 (Low)	Fifth- to sixth-year medical students from 36 Latin American universities.
Hassan (2023)	2023	Sudan	Probabilistic	Self-report	72/149	48.32%	87 (58.4%)	Not reported	8 (Low)	First- to sixth-year medical students.
Wang (2023)	2023	United States	Non-probabilistic	Self-report	120/154	77.92%	Not reported	Not reported	7 (Low)	Medical students.
Bhatnagar (2023)	2023	India	Non-probabilistic	Self-report	60/283	21.20%	130 (45.94%)	21.67	7 (Low)	Medical students using digital devices for at least one month.
Lulla (2023)	2023	India	Non-probabilistic	OSDI	148/271	54.61%	Not reported	Not reported	8 (Low)	Volunteer medical students; chemical injuries or corneal surgery excluded.
Utlu (2024)	2024	Turkey	Non-probabilistic	OSDI	163/255	63.92%	192 (75.3%)	20	8 (Low)	Volunteer medical students aged 18-25 years; smokers, chronic systemic disease, topical ocular medication, or no consent excluded.
Abu-Ismail (2024)	2024	Jordan	Non-probabilistic	OSDI	905/1223	74.00%	738 (64.0%)	Not reported	8 (Low)	Medical students.
Almousa (2024)	2024	Saudi Arabia	Non-probabilistic	Self-report	204/300	68.00%	176 (58.7%)	21.5	7 (Low)	First- to fifth-year medical students; ocular pathology, recent surgery, systemic disease, and medications with visual effects excluded.
Aljammaz (2021)	2021	Saudi Arabia	Probabilistic	McMonnies questionnaire	45/247	18.22%	176 (71.25%)	Not reported	9 (Low)	First- to sixth-year medical students and interns older than 18 years; no consent excluded.
Basnet (2024)	2024	Nepal	Non-probabilistic	OSDI	68/110	61.82%	43 (39.09%)	30.4	8 (Low)	Medical students; incomplete forms or peripheral posts excluded.
Preoteasa (2024)	2024	Romania	Non-probabilistic	OSDI	229/274	83.58%	223 (81.4%)	22.7	8 (Low)	Medical students aged 18-38 years; language barrier, recent eye surgery, eye infection, and systemic disease excluded.
Kunboon (2024)	2024	Thailand	Non-probabilistic	DEQS-Th	271/449	60.36%	61.5%	21.8	8 (Low)	Medical students older than 18 years; chronic ocular disease, recent infection/surgery, systemic disease, and psychological disorder excluded.
Alkousheh (2024)	2024	Jordan	Non-probabilistic	Self-report	567/833	68.07%	543 (65.2%)	21	7 (Low)	First- to sixth-year medical students; other programs or no consent excluded.
